# Improved Pancreatic Adenocarcinoma Diagnosis in Jaundiced and Non-Jaundiced Pancreatic Adenocarcinoma Patients through the Combination of Routine Clinical Markers Associated to Pancreatic Adenocarcinoma Pathophysiology

**DOI:** 10.1371/journal.pone.0147214

**Published:** 2016-01-25

**Authors:** María José Ferri, Marc Saez, Joan Figueras, Esther Fort, Miriam Sabat, Santiago López-Ben, Rafael de Llorens, Rosa Núria Aleixandre, Rosa Peracaula

**Affiliations:** 1 Clinic Laboratory, Dr. Josep Trueta University Hospital, Girona, Spain; 2 Research Group on Statistics, Econometrics and Health (GRECS), University of Girona, Girona, Spain; 3 CIBER of Epidemiology and Public Health (CIBERESP), Girona, Spain; 4 Hepato-biliary and Pancreatic Surgery Unit, Dr. Josep Trueta University Hospital, Girona Biomedical Research Institute (IDIBGI), Girona, Spain; 5 Gastroenterology Unit, Dr. Josep Trueta University Hospital, Girona, Spain; 6 Gastroenterology Unit, Hospital Santa Caterina, Salt, Girona, Spain; 7 Department of Biology, University of Girona, Girona, Spain; University of Ulm, GERMANY

## Abstract

**Background:**

There is still no reliable biomarker for the diagnosis of pancreatic adenocarcinoma. Carbohydrate antigen 19–9 (CA 19–9) is a tumor marker only recommended for pancreatic adenocarcinoma follow-up. One of the clinical problems lies in distinguishing between this cancer and other benign pancreatic diseases such as chronic pancreatitis. In this study we will assess the value of panels of serum molecules related to pancreatic cancer physiopathology to determine whether alone or in combination could help to discriminate between these two pathologies.

**Methods:**

CA 19–9, carcinoembryonic antigen (CEA), C-reactive protein, albumin, insulin growth factor-1 (IGF-1) and IGF binding protein-3 were measured using routine clinical analyzers in a cohort of 47 pancreatic adenocarcinoma, 20 chronic pancreatitis and 15 healthy controls.

**Results:**

The combination of CA 19–9, IGF-1 and albumin resulted in a combined area under the curve (AUC) of 0.959 with 93.6% sensitivity and 95% specificity, much higher than CA 19–9 alone. An algorithm was defined to classify the patients as chronic pancreatitis or pancreatic cancer with the above specificity and sensitivity. In an independent validation group of 20 pancreatic adenocarcinoma and 13 chronic pancreatitis patients, the combination of the four molecules classified correctly all pancreatic adenocarcinoma and 12 out of 13 chronic pancreatitis patients.

**Conclusions:**

Although this panel of markers should be validated in larger cohorts, the high sensitivity and specificity values and the convenience to measure these parameters in clinical laboratories shows great promise for improving pancreatic adenocarcinoma diagnosis.

## Introduction

Pancreatic ductal adenocarcinoma (PDAC) is the fourth leading cause of cancer-related mortality in the United States. Data show that pancreatic cancer has hardly improved in terms of survival in recent years in relation to other carcinomas [1]. The poor prognosis of pancreatic cancer is mainly due to the advanced stage of the disease at the time of diagnosis and to the aggressive nature of the tumor.

Although imaging and invasive endoscopic technologies are becoming increasingly accurate, early diagnosis is still difficult [2–3]. Existing tumor markers are not sufficiently specific as to be able to distinguish between benign and malignant forms of the disease and there is particular difficulty in the differential diagnosis of chronic pancreatitis (CP) from PDAC. Imaging techniques such as FDG-PET did not help in the distinction of pancreatic cancer from focal mass-forming pancreatitis, which was FDG-PET positive in 79% of cases [4]. Various types of chronic pancreatitis can closely mimic pancreatic cancer both clinically and morphologically especially in small biopsy specimens [5]. A known interference in tumor imaging diagnosis is the desmoplastic stroma reaction induced by the tumor in the surrounding soft tissue [6]. The unreliability of the available diagnostic tools has led to 10% of cases receiving unnecessary surgery after being erroneously classified as having pancreatic cancer [7]. A new non-invasive biomarker that discriminates between CP and PDAC would be of great value in dealing with this diagnostic deficiency.

The two most studied tumor markers that have been evaluated in PDAC are carbohydrate antigen 19–9 (CA 19–9) and carcinoembryonic antigen (CEA). CA 19–9 is widely used in the management of PDAC but it has serious limitations: it has a specificity of 82% (68–91%) and a sensitivity of 79 (70–90%) [8]. It shows high levels in other malignancies and in benign gastrointestinal diseases such as CP which makes it not suitable for PDAC diagnosis.

The current limitations in PDAC diagnosis have made it necessary to search for new biomarkers. Microarrays and proteomics analysis have identified genes and proteins highly expressed in PDAC, which have then been tested as potential PDAC tumor markers, but they have not been found to perform better than CA 19–9 [9–14]. The failure of single serum biomarkers could be explained by the complex biology of PDAC, and this has recently led several groups to examine the use of a combination of different factors related with pancreatic cancer pathophysiology.

Thus, some studies have combined CA 19–9 with other markers in order to improve sensitivity and specificity in PDAC diagnosis. Biomarker profiles include factors produced by the tumor and factors from the systemic response to the growing tumor, including inflammatory reactants. They show that a combination of biomarkers offers an improved diagnostic ability [7,15–17]. However, the fact that the described increases in sensitivity and specificity are not always much higher than those of CA 19–9 alone and that some of the molecules assayed are not routinely analyzed in the clinical laboratories limits the clinical translation of the reported combinations. In addition some features linked to PDAC such as obstructive jaundice, which is associated with tumors involving the pancreatic head, have been reported to influence in the performance of biomarkers [3,18–19].

In this study we have analyzed the serum levels of several molecules related to PDAC pathophysiology in order to determine possible combinations that could help to improve the differential diagnosis of PDAC from CP. Apart from CA 19–9 and CEA tumor markers, we have analyzed different molecules related with the inflammatory component and other molecules associated with the IGF (insulin growth factor) axis, which are related to PDAC pathophysiology. In particular, we have quantified the serum levels of two acute-phase proteins C-reactive protein (CRP) as a positive inflammation marker and albumin as a negative acute-phase reactant, and IGF-1 and IGFBP3 serum levels have been measured as parameters indicative of the IGF-1 axis. We have also assessed the potential effect of obstructive jaundice in the performance of the markers.

CRP and albumin were the acute phase proteins measured since both markers constitute the Glasgow prognostic score, which is a cumulative score based on elevated CRP and hypoalbuminemia. This score has been reported to be of prognostic value in different types of cancers including PDAC [20–21]. Regarding the IGF-1 axis, previous studies have shown that higher levels of IGF-1 and low levels of IGFBP3 are associated with an enhanced risk of PDAC [22–24], although other studies did not find this relationship [25–26]. Few studies have analyzed IGF-1 and IGFBP3 serum levels in PDAC and CP patients and their results show discrepancies probably due to the low number of patients analyzed and the different methodologies used [27–29].

In this study, the value of the serum tumor markers CA19.9, CEA, CRP, albumin, IGF-1 and IGFBP3 alone or in combination, in the differential diagnosis of PDAC and CP was assessed. The molecules chosen are routinely analyzed in clinical laboratories using commercial kits, which would facilitate the potential clinical translation of this approach and could constitute a non-invasive test in the differential diagnosis of these two pancreatic diseases.

## Material and Methods

### Patient and serum samples

This study was approved by the Ethics Committee of the Dr. Josep Trueta University Hospital and informed written consent was obtained from all participants.

From 2007 to 2012 serum samples from 47 PDAC patients (mean age (SD) 64.1 (10.3), 28 male, 19 female), 20 CP patients (mean age (SD) 56.3 (11.5), 16 male, 4 female) and 15 healthy controls (HC) (mean age (SD) 60.9 (9.2), 4 male, 11 female) were obtained from the Dr. Josep Trueta University Hospital (Table 1). The PDAC patients were subdivided in two groups, PDAC1 and PDAC2, depending on being non-jaundiced and jaundiced patients, respectively. PDAC jaundiced patients were 30 patients with bilirubin levels above 2 mg/dL (mean of 13.77 mg/dL, range 2.5–26 mg/dL), which corresponds to 64% of the individuals of the PDAC group. PDAC non jaundiced patients had bilirubin levels below 2 mg/dL (mean of 0.82 mg/dL, range 0.4–1.9 mg/dL).

**Table 1:** Clinical and pathological characteristics of the patients.

			PDAC	
	HC	CP	PDAC 1	PDAC2
**N**	15	20	17	30
**Age median**	60.9	56.3	66.3	62.8
**Male**	4	16	9	19
**Female**	11	4	8	11
**BMI median**	25.3	23.4	26.3	25.7
**Stage IA**			0	0
**Stage IB**			1	0
**Stage IIA**			1	3
**Stage IIB**			7	15
**Stage III**			5	3
**Stage IV**			3	9

PDAC: pancreatic ductal adenocarcinoma; PDAC1: Bilirubin < 2 mg/dL; PDAC2: Bilirubin > 2 mg/dL; CP: chronic pancreatitis; HC: healthy controls.

An independent set of samples for validation purposes comprised 20 PDAC patients (mean age 67.2 (SD 10.4); 9 male, 11 female) and 13 chronic pancreatitis patients (mean age 50.9 (SD 9.55); 10 male, 3 female) (Table 2). From the PDAC patients 35% were non-jaundiced and 65% were jaundiced individuals. The jaundiced group had bilirubin levels within the range 2–12.8 mg/dL (mean of 8.61 mg/dL) and the non-jaundiced group had bilirubin levels within the range 0.4–1.9 mg/dL (mean of 1.17 mg/dL).

**Table 2:** Clinical and pathological characteristics of the validation set.

		PDAC	
	CP	PDAC 1	PDAC2
**N**	13	7	13
**Age median**	50.9	66.4	67.6
**Male**	10	4	5
**Female**	3	3	8
**Stage IA**		0	0
**Stage IB**		0	0
**Stage IIA**		1	6
**Stage IIB**		5	4
**Stage III**		0	1
**Stage IV**		1	2

PDAC: pancreatic ductal adenocarcinoma; PDAC1: Bilirubin < 2 mg/dL; PDAC2: Bilirubin > 2 mg/dL; CP: chronic pancreatitis.

Samples from cancer patients were taken by venipuncture before surgical intervention. All sera were collected using standard procedures, aliquoted to avoid repeated freeze thaw cycles and stored at -80°C until analysis.

Diagnosis of PDAC patients was derived from the histopathologic examination of the pancreaticoduodenectomy in resectable tumours and with cross-sectional images and percutaneous or endoscopic biopsy in unresectable ones and diagnosis of CP was based on standard radiological imaging, pancreatic functional tests and clinical evolution. Patients that had been diagnosed with other malignancies in the last five years were excluded as were CP patients with an acute disease flare-up.

### Assay methods

All parameters were measured in automatic systems and with standardized reagents used in clinical practice routine at the hospital laboratory.

The analysis of CA 19–9 and CEA were determined with the quantitative electrochemiluminescence immunoassay intended for use with the E-170 (Roche Diagnostics). The CA 19–9 assay is based on the monoclonal 116-NS 19–9 antibody. The albumin and bilirubin assays were performed with colorimetric assays in a Cobas 711 (Roche). The quantitative determination of CRP was measured by particle enhanced immunoturbidimetric assay in the Cobas 711,IGF-1, IGFBP3 and Growth Hormone (GH) were determined using an Immulite 2000 analyzer (Siemens) with the quantitative measurement solid-phase-enzyme-labeled chemiluminiscent immunometric assay

Nephelometry was used to measure prealbumin with an Immage 800 (Beckman),

### Statistics

Data analysis was performed with the SPSS statistical package v 15.0. Patient characteristics were compared between pancreatic cancer, chronic pancreatitis an healthy controls using the U Mann-Whitney test. For all univariate analysis, pancreatic ductal adenocarcinoma was considered the disease state. The Spearman’s rank correlation test was applied to assess the correlation between two variables.

The statistical software R version 3.1.0 [30] was used for ROC (receiver operating characteristic) analysis. ROC curves were used to describe the performance of biomarkers or the performance of the combination of different markers as a diagnostic test. The area under the curve (AUC) was used to evaluate the usefulness of a biomarker as a diagnostic test and the cut-off levels were calculated [31–32]. To combine markers, a logistic regression was specified, in which the response variable was the probability that the event of interest was pancreatic cancer (variable taking the value 1) or chronic pancreatitis (variable taking the value 0). As explanatory variables, in addition to the marker CA19.9, the other markers were included in the equation as predictors (one to one). The optimal combination of markers was the one that provided the greatest AUC of the ROC curve.

The generalized lineal model (GLM) with binomial response was estimated with the R statistical package. The Epi [33–34] and pROC libraries [35], also in the R platform, were used for the construction and comparison respectively of the AUC of the different ROC curves. Significance was set at p<0.05.

## Results

### Clinical characteristics of study population

Serum samples of 82 subjects; 47 PDAC, 20 CP and 15 HC, were analyzed. The 47 PDAC patients TNM histological distribution included different stages (stage IB n = 1; IIA n = 4; IIB n = 22, III n = 8; IV n = 12) according to the International Union Against Cancer (UICC) staging [36] and of these 29 had resectable tumors and 30 were jaundiced.

The age, gender and the body mass index (BMI) of the patients are shown in Table 1. While the PDAC and HC groups did not show significant differences in age, gender and BMI, the CP group tended to be formed by males with a lower BMI than the other two groups.

### Univariate analysis of biomarkers: tumor markers (CA 19–9 and CEA), acute-phase proteins (albumin and CRP) and IFG-1 and IGFBP3 in PDAC, CP and HC groups

**Fig 1:**
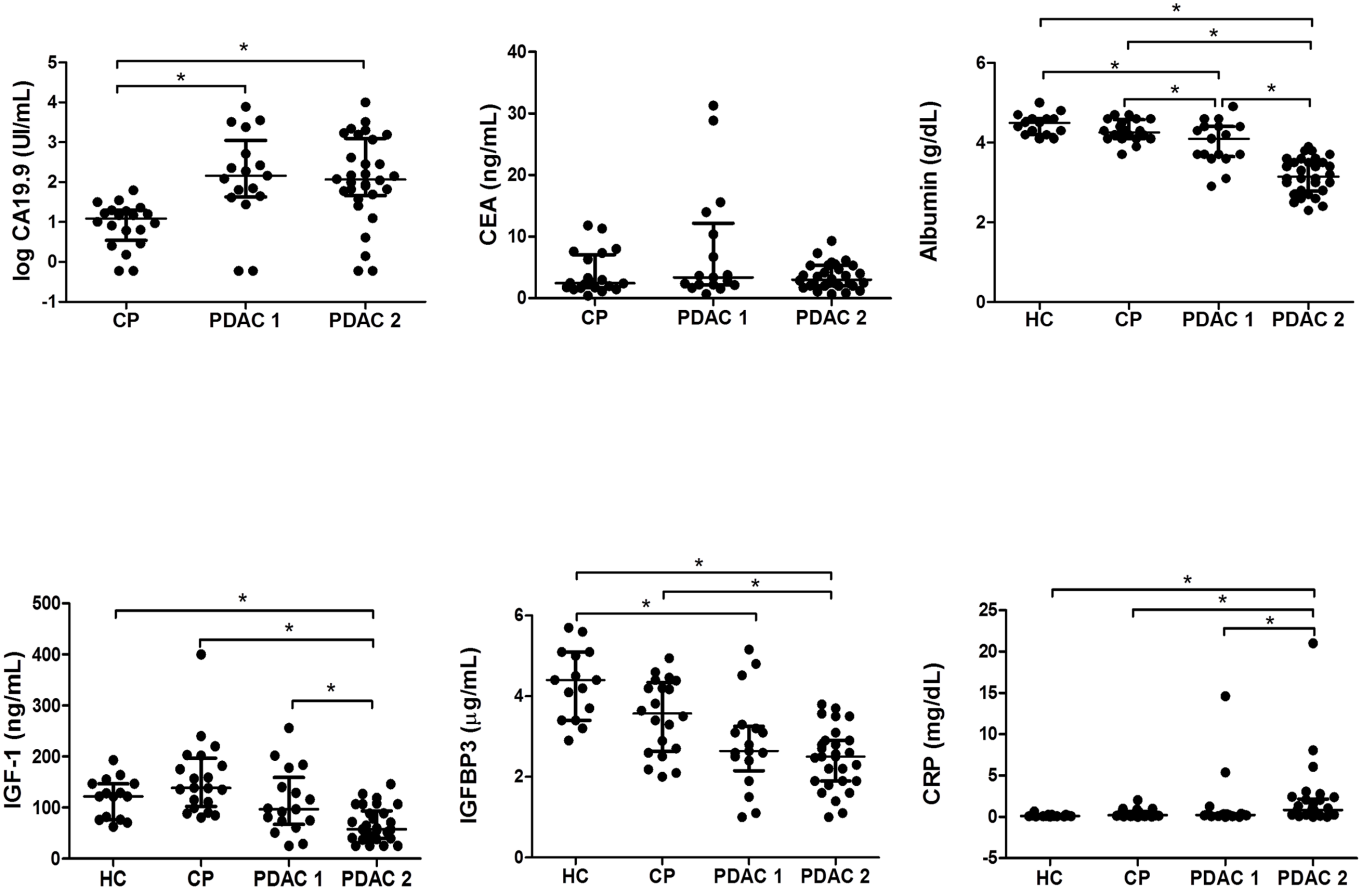
Scatter dot plots of serum levels of CA 19–9, CEA, albumin, C-reactive protein, IFG-1 and IGFBP3 in non-jaundiced and jaundiced pancreatic ductal adenocarcinoma (PDAC 1 andPDAC2, respectively), chronic pancreatitis (CP) and healthy control (HC) groups. The values of individual sera are represented with dots. The center line in the box represents the median, and the top (Q3) and bottom (Q1), the 75^th^ and 25^th^ percentiles, respectively.

Serum levels of each molecule were compared between PDAC, CP and HC groups and also separating the PDAC group into non-jaundiced (PDAC1) and jaundiced patients (PDAC2). They are shown in scatter plots (Fig 1).

CA 19–9 and CEA were only measured in the chronic pancreatitis and pancreatic cancer groups since the level of these tumor markers in HC are reported to be clearly below the established cut-offs [10,17,37]. To assess their specificity and sensitivity, ROC curves were performed for all of them.

#### CA 19–9 and CEA

The mean serum concentration of CA 19–9 in pancreatic cancer patients was 945 U/mL, significantly higher than in chronic pancreatitis patients (15.1 U/mL) (p<0.001).

For clarity, log10 of CA19.9 levels were plotted. Using a cut off of 37 U/mL (log10 CA19.9>1.56), the specificity was 95%, the sensitivity 80.9% and the ROC curve gave an AUC of 0.862.

CA19.9 levels were found above 37U/mL in 80,9% of PDAC patients. 64% of PDAC presented jaundice with total bilirubin levels above 2mg/dL. Plots of the PDAC non-jaundiced patients (PDAC1) and jaundiced patients (PDAC2) are shown. No significant correlation between high CA19.9 levels and total bilirubin levels was found in this cohort of patients. The jaundiced PDAC patients that showed CA19.9 levels above 37 U/mL were 80% and the non-jaundiced PDAC patients were 82,4%. No significant differences in CA19.9 levels were detected between PDAC1 (mean serum concentration of 1104 U/mL) and PDAC2 (mean serum concentration of 856 U/mL) groups. CA19.9 levels could significantly differentiate between each PDAC group, PDAC1 and PDAC2, from the CP group (p = 0.001 and p<0.001, respectively).

CEA serum levels did not show significant differences between CP and any of the PDAC groups.

#### CRP and albumin

The mean serum concentration of CRP in PDAC patients was 18 mg/L, significantly higher than in CP patients (4 mg/L) and in HC (1.69 mg/L) (p = 0.038; p = 0.019). Using a cut off of 2.3 mg/L, the specificity was 55% and the sensitivity 76.6%. ROC curve for differentiating PDAC from CP gave an AUC of 0.661.

CRP levels of PDAC1 (mean of 14.19 mg/L) and PDAC2 (mean of 18.86 mg/L) were significantly different (p = 0.012). CRP levels from PDAC 2 were significantly higher than CP (4mg/L) and HC (1.69 mg/mL) groups (p = 0.004 and p<0.001, respectively). However, CRP levels of the PDAC1 group were not significantly different from the CP and HC groups.

The mean serum concentration of albumin in PDAC patients was 34 g/L significantly lower than in CP patients (43 g/L) and in HC (45 g/L), (p<0.001 and p<0.001, respectively). Using a cut off of 38 g/L, the specificity was 95% and the sensitivity 78.7%. ROC curve for differentiating PDAC from CP gave an AUC of 0.870.

Albumin levels of PDAC1 (median of 39 g/L) and PDAC2 (median of 32 g/L) were significantly different (p = 0.012). However, in both PDAC groups (PDAC1 and PDAC2) albumin levels were significantly lower than in CP patients (p = 0.046 and p<0.001, respectively) and in HC patients (p = 0.07 and p<0.001, respectively) Jaundice is influencing in both albumin and CRP levels in PDAC patients. In the case of albumin levels, however, the non-jaundiced PDAC patients (PDAC1) still showed significant differences with respect to CP and HC patients.

#### IGF-1 axis: IGF-1, IGFBP3 and growth hormone (GH)

IGF-1 serum levels did not show differences between HC and CP patients but there were differences between the HC and PDAC patients (p = 0.002) and the CP and PDAC groups (p<0.001).

Using a cut off of 80.7 ng/mL, the specificity was 100% and the sensitivity 57.4%. ROC curve for differentiating PDAC from CP gave an AUC of 0.839.

IGF-1 levels of PDAC1 (mean of 111 ng/mL) and PDAC2 (mean of 67.1 ng/mL) were significantly different (p = 0,012). IGF-1 levels from PDAC 2 were significantly lower than CP and HC (p<0.001 and p<0.001). However, the IGF-1 levels of the PDAC1 group were not significantly different from the CP and HC groups.

IGFBP3 serum showed differences between HC and CP, HC and PDAC and CP and PDAC groups (p = 0.023; p<0.001 and p = 0.002, respectively).

Using a cut off of 3.34 mcg/mL, the specificity was 60% and the sensitivity 82.2%. ROC curve for differentiating PDAC from CP groups gave an AUC of 0.734.

IGFBP3 levels of PDAC1 (median of 2.85 mcg/mL) and PDAC2 (median of 2.45 mcg/mL) were not significantly different between them. IGFBP3 levels from PDAC2 were significantly lower than CP and HC (p<0.001 and p<0.001). IGFBP3 levels from PDAC1 were only significantly lower than the HC group (p<0.001).

To determine whether the lower IGF-1 levels found in general in PDAC patients could be associated to either a malnutrition status or to growth hormone resistance, prealbumin and GH levels were measured as the respective indicators of the two disorders as well as their possible correlation with IGF-1 levels.

Although prealbumin levels were lower in the PDAC than in the CP and HC groups, the mean concentration of prealbumin did not show significant differences between PDAC (mean 0.165 g/L, SD 5.6) and CP (mean 0.199 g/L, SD 4.4), but there were significant differences between CP and HC (mean 0.255 g/L, SD 3.84) and between PDAC and HC. IGF-1 and prealbumin levels showed a positive correlation (r = 0.454, p = 0.005) in the PDAC group but not in the CP group.

Concerning GH levels, there were no significant differences between PDAC (mean 1.69 ng/mL, SD 2.16) and CP (mean 1.58 ng/mL, SD 1.71) or HC (mean 1.57, SD 1.81) groups. No correlation was found between IGF-1 and GH levels.

Since the CP group presented a bias to male cases, we also examined whether the gender could influence in the behaviour of the above markers in the CP group. Statistical analysis did not show any significant difference in the levels of the markers except for CRP that in the CP female group showed lower serum values than in the male group.

In Table 3, the sensitivity, specificity, negative predictive value (NPV), positive predictive value (PPV) and AUC of each marker for differentiating PDAC from CP groups are shown. As shown, no single marker performed significantly better than CA 19–9, but as mentioned above, CA 19–9 is not sufficiently sensitive and specific to be used for differential PDAC diagnosis.

**Table 3:** Diagnostic parameters of the different single markers to differentiate pancreatic cancer from chronic pancreatitis.

	Sensitivity (%)	Specificity (%)	PPV^a^ (%)	NPV^b^ (%)	AUC^c^
**CA 19–9**	80.9	95	97.4	67.9	0.862
**CEA**	83	40	76.5	50	0.561
**Albumin**	78.7	95	97.4	65.5	0.869
**CRP**	76.6	55	80	50	0.654
**IGF-1**	57.4	100	100	50	0.839
**IGFBP3**	82.2	60	82.2	60	0.734

^a^PPV, positive predictive value,

^b^NPV, negative predictive value,

^c^AUC area under the Roc curve.

### Multivariate analysis of biomarker levels

In order to assess the performance of the combination of the different biomarkers, the R statistic package was used. The sensitivity, specificity, NPV, PPV and AUC of each combination of biomarkers for differentiating PDAC from CP groups were evaluated (Table 4).

**Table 4:** Diagnostic parameters of the different combinations of markers to differentiate pancreatic adenocarcinoma from chronic pancreatitis.

	Sensitivity(%)	Specificity (%)	PPV^a^ (%)	NPV^b^ (%)	AUC^c^
CA 19–9	80.9	95	97.4	67.9	0.862
CA 19–9+CEA	83	95	97.5	70.4	0.887
CA 19–9+IGF-1	83	100	100	71.4	0.946
CA 19–9+Albumin	95.7	95	97.8	90.5	0.954
CA19-9 +CRP	83	95	97.5	70.4	0.907
CA 19–9+ IGF-1+CEA	80.9	100	100	69	0.947
**CA 19–9+ IGF-1+Albumin**	**93.6**	**95**	**97.8**	**86.4**	**0.959**
CA 19–9+IGF-1+CEA+Albumin 1+CEA+Albumin	97.9	95	97.9	95	0.979
CA 19–9+CEA+IGF-1+CRP	87.2	100	100	76.9	0.954
CA 19–9+CEA+IGF-1+Albumin+CRP	91.5	100	100	83.3	0.979

^a^PPV, positive predictive value,

^b^NPV, negative predictive value,

^c^AUC area under the Roc curve.

Taking CA 19–9 as a reference, the best combination of two biomarkers was that of CA 19–9 and IGF-1, which gave an AUC of 0.946, much higher than CA 19–9 alone.

Adding a new biomarker such as CEA slightly improved the performance to 0.947. When adding albumin, the AUC increased to 0.979. No improvement was shown when adding either CRP or IGFBP3 to the combination.

**Fig 2:**
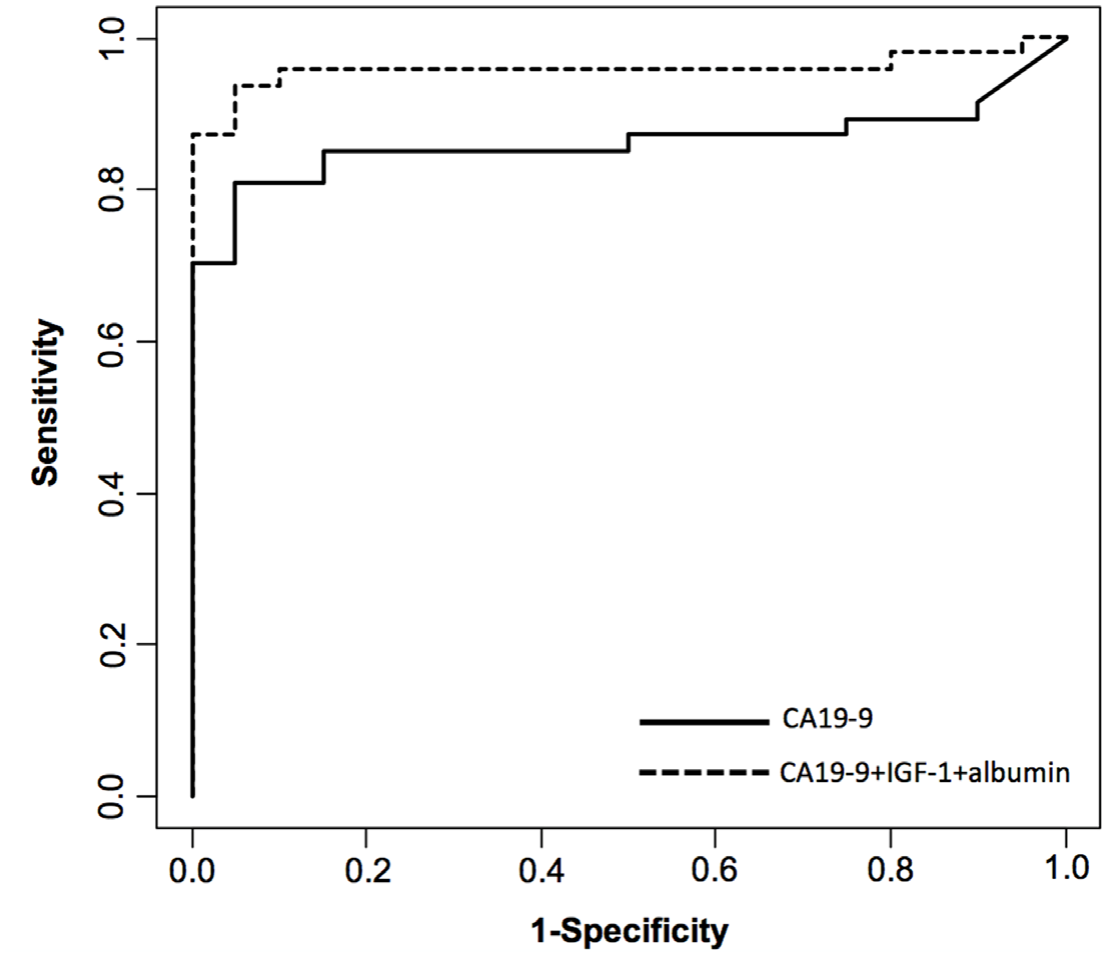
Receiver operator characteristic (ROC) curve for diagnosis of pancreatic cancer versus chronic pancreatitis. Diagnostic performance of CA 19–9, IGF-1 and albumin combination (dotted line) compared with CA 19–9 alone (solid line).

As CEA alone was not discriminatory, we assayed the combination without CEA to test whether this marker was necessary to be included in the model. The combination of CA 19–9, IGF-1 and albumin gave a slightly lower AUC (0.959) than when combining CA 19–9, CEA, IGF-1 and albumin (AUC of 0.979). The limited number of CP patients of this cohort suggests that four variables could lead to overfitting of the model [38], and as CEA alone was not discriminatory, a model without including CEA has been used in this study to avoid possible overfitting. Thus, the combination of the following three markers: CA 19–9, IGF-1 and albumin was used to distinguish PDAC from CP patients with 93.6% sensitivity and 95% specificity (Fig 2).

Since some of the individual markers performed better in the jaundiced patients (PDAC2 group), in particular those that were liver derived such as albumin, CRP, IGF-1 and IGFBP3, we analyzed the potential of the combination of CA 19–9, IGF-1 and albumin for discriminating each PDAC group from CP. The jaundiced group (PDAC2) showed better discrimination than the non-jaundiced one (PDAC1) (AUC of 1 *vs*. AUC of 0.903, with 88.2% sensitivity and 90% specificity), suggesting that probably few markers would be enough to discriminate the jaundiced PDAC from CP. Nevertheless, this combination of markers allows distinguishing the overall PDAC patients, composed of PDAC1 and PDAC2 patients, from CP with high performance (AUC 0.959).

In order to implement this combination of biomarkers in clinical practice, an algorithm consisting of a generalized lineal model with a binomial response was designed. After introducing CA 19–9, IGF-1 and albumin serum levels, the GLM classifies the patients as having PDAC with 93.6% sensitivity and 95% specificity. The cut-offs for each parameter are: CA 19–9 62.7 U/mL; IGF-1 136 ng/mL and albumin 46 g/L. With this model the probability of new patients being diagnosed as CP or PDAC was calculated. For a probability equal to or higher than 51.3% (point with maximum sensitivity and specificity) the patient will be classified as PDAC with a sensitivity of 93.6%, but if the probability is lower than 51.3% the patient is classified as CP with a specificity of 95%. The probability of being classified as CP or PDAC for a particular patient is calculated with their values of CA 19–9, IGF-1 and albumin, using the following function, where β0, β1, β2, β3 and β4 are parameters estimated by the model:

$$\text{Pr}ob\left(Pancreatic\_cancer\right)=\frac{e^{\left(\beta_{0}+\beta_{1}CA19-9+\beta_{2}IGF1+\beta 3Albu\text{min}\right)}}{1+e^{\left(\beta_{0}+\beta_{1}CA19-9+\beta_{2}IGF1+\beta 3Albu\text{min}\right)}}$$

The panel of CA 19–9, IGF-1 and albumin was validated in an independent validation set, composed of 20 PDAC and 13 CP patients (Table 2). The TNM distribution of the 20 PDAC samples was: stages IIA n = 7; IIB n = 9; III n = 1 and IV n = 3, of these 16 were resectable tumors and 65% of PDAC patients were jaundiced.

**Fig 3:**
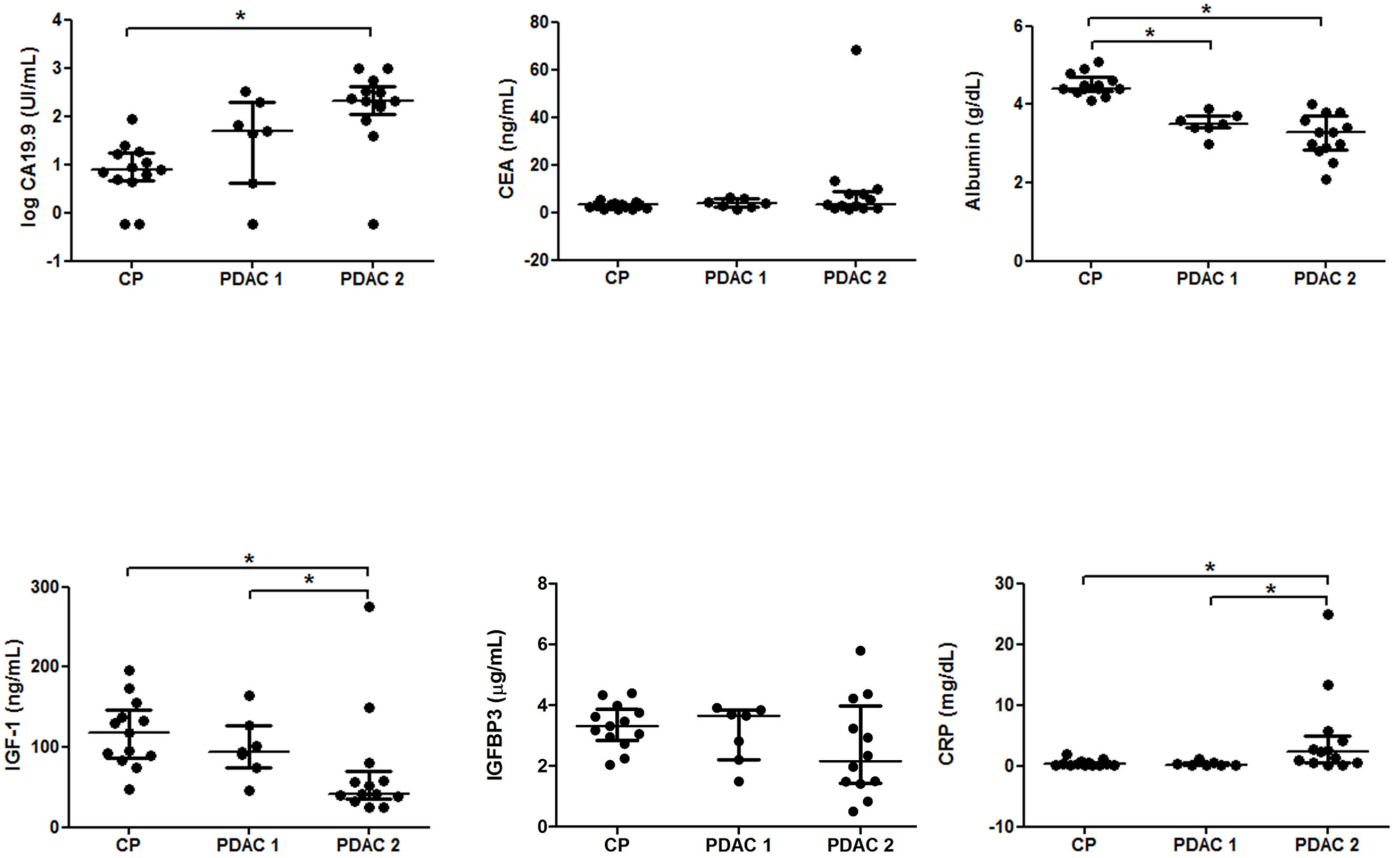
Scatter dot plots of serum levels of CA 19–9, CEA, albumin, C-reactive protein, IFG-1 and IGFBP3 in non-jaundiced and jaundiced pancreatic ductal adenocarcinoma (PDAC 1 and PDAC2, respectively) and chronic pancreatitis (CP) groups. The values of individual sera are represented with dots. The center line in the box represents the median, and the top (Q3) and bottom (Q1), the 75^th^ and 25^th^ percentiles, respectively.

Plots of the univariant markers for PDAC1 (non-jaundiced), PDAC2 (jaundiced) and CP patients of this validation set were analyzed (Fig 3). They showed a similar behavior than those of the previous set of patients analyzed. CA19-9 levels showed significant differences between PDAC2 and CP groups as before (p<0.001), but unlike the other set of patients, CA19-9 levels of PDAC1 patients were not found significantly higher than the ones of the CP group, suggesting that in this set of patients jaundice could be influencing in elevating CA19-9 levels. However, no significant correlation between CA19.9 levels and total bilirubin levels was found in the PDAC patients and no significant differences in the CA19.9 levels between PDAC groups were either found. CEA levels did not show any significant differences among the groups. CRP levels were significantly different between PDAC1 and PDAC2 (p = 0.008) and only PDAC2 showed significantly higher CRP levels than CP (p = 0.008). Both PDAC1 and PDAC2 albumin levels were significantly lower than the levels of the CP group (p<0.001) as in the previous set of patients. IGF-1 levels were also significantly lower in the PDAC patients than in CP (p = 0.012) and significant differences between PDAC groups were also found (p = 0.029). IGF-1 levels of PDAC2 showed also a significant decrease compared to CP (p = 0.004) but not the ones of the PDAC1 group, as in the previous set of patients. Finally, regarding IGFBP3 levels, significant differences between groups were not detected in this set.

**Fig 4:**
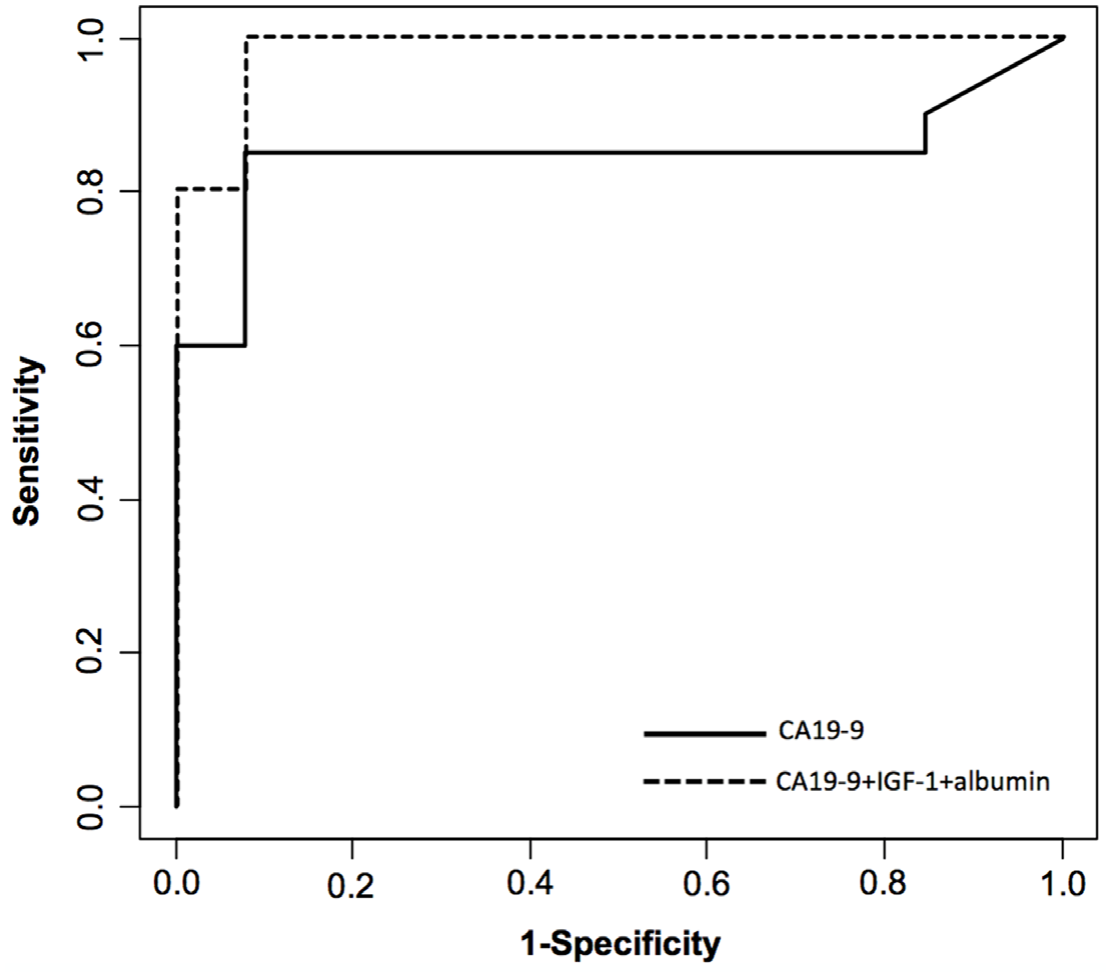
Receiver operator characteristic (ROC) curve for diagnosis of pancreatic cancer versus chronic pancreatitis in the validation set of patients. Diagnostic performance of CA 19–9, IGF-1 and albumin combination (dotted line) compared with CA 19–9 alone (solid line).

Each validation sample was diagnosed as cancer or chronic pancreatitis using the above described function. Using this model, 20 out of 20 PDAC patients and 12 out of 13 CP patients were correctly classified, which corresponds to 100% sensitivity and 92.3% specificity. The ROC curve applying this model is shown in Fig 4.

## Discussion

The lack of available serum biomarkers for PDAC diagnosis and the complex biology of this disease are leading the scientific community to search for a combination of potential molecules linked to PDAC rather than for a single molecule as potential new PDAC biomarkers. The importance of the differential diagnosis between CP and PDAC remains still a clinical challenge [7,39]. In addition, since the effect of jaundice in the performance of biomarkers has recently been reported [3,18–19], it has also been examined in this work separating the group of PDAC patients into jaundiced and non-jaundiced individuals.

Here, we have assessed how the combination of several molecules associated to several aspects of PDAC physiopathology, such as the inflammatory component and the growth factor axis, together with the tumor markers CA 19–9 and CEA could differentiate between PDAC and CP. We evaluated the performance in differentiating PDAC from CP by combining the new molecules with CA 19–9, which is used in following up PDAC. In our cohort of patients, the best combination of three markers was achieved with CA 19–9, IGF-1 and albumin with 93.6% sensitivity, 95% specificity and an AUC of 0.959. The validation of this methodology could be corroborated with an independent set of PDAC and CP samples. Even though the validation set had a limited number of cases, the biomarker trends were similar than those of the discovery set and using the algorithm that combines the three markers 100% sensitivity and 92.3% specificity were obtained, in line with the above reported values.

With regards to the acute-phase proteins evaluated, CRP and albumin, the former did not contribute to improving the differential diagnosis between PDAC and CP. Miyake et al. showed that high-sensitivity-CRP (hs-CRP) can be useful in the diagnosis of PDAC [40]. However, in their study they compared HC and PDAC patients while in our study we have compared PDAC and CP, which both presented higher inflammation in relation to controls. This could explain why neither hs-CRP nor CRP serum values led to an improvement in PDAC diagnosis in our study. CRP values of PDAC patients were also influenced by jaundice, and only the group of jaundiced PDAC patients showed significantly higher levels than the CP and HC groups.

The inclusion of albumin contributed to improving the performance of the combination of markers, increasing the sensitivity rather than the specificity. Significant lower albumin levels were found in both groups of PDAC patients as shown in Figs 1 and 3, which could be due to a combination of inflammation and malnutrition in advanced cancer. The CP group generally showed low BMI values, which could in part be associated with a malnutrition state, although the albumin values of the CP group did not differ significantly with respect to the controls.

Concerning the IGF-1 axis, serum IGFBP3 levels did not contribute to improving the performance of the combination of markers, while serum IGF-1 levels entailed a substantial improvement in specificity. Significantly lower levels of IGF-1 were found in the jaundiced PDAC than in CP or HC patients (Figs 1 and 3). The reduction of serum IGF-1 levels could be associated to an impaired hepatic function and patient’s nutritional status, which in the case of cancer patients involves weight loss [41]. In this regard, in the PDAC group IGF-1 levels correlated with low prealbumin levels (p = 0.005), which are indicative of the nutritional status. This correlation was not found in the CP group. On the other hand, Okamoto et al. described an alteration of the GH-IGF-1 axis, involving high GH levels and low IGF-1 levels in several cancer patients [42]. In our PDAC group we did not find a significant correlation between GH and IGF-1 levels.

The performance of this combination of markers for pancreatic cancer diagnosis is better or similar to that found in several recent studies. Balasethil et al. obtained an AUC near to 1 with a combination of three plasma biomarkers Tenascin C, tissue factor pathway inhibitor and CA 19–9 but they only compared PDAC patients with HC and these two groups are not challenging for differential diagnosis [37]. Koopman et al. comparing PDAC and CP patients obtained an AUC of 0.84 with the combination of macrophage inhibitory cytokine-1 and CA 19–9 [9]. Recently, Zhang et al, described that the combination of CA19.9, CO_2_, CRP and IL-6 distinguish pancreatic cancer from benign pancreatitis with 74.2% sensitivity at 90% specificity [16]. Chung et al. described that a multi-marker panel composed of four molecules, uric acid, soluble MHC class I chain-related molecules A and B, and CA 19–9 detect pancreatic cancer from non-cancerous conditions (healthy controls and chronic pancreatitis) with 94.2% sensitivity and 93.3% specificity [43]. In all cases the reported combinations achieved a superior diagnostic than CA19.9 alone. Zeh et al. used a panel of 10 cytokines and achieved also an improvement of sensitivity and specificity versus CA 19–9, but this panel combination requires special equipment to be translated into clinical practice [7]. Faca et al. used a group of 7 proteins associated to pancreatic cancer and discriminated PDAC from CP with an AUC value of 0.96 [17]. The use of a large panel of molecules that are not included in the test menu of routine laboratory limits the applicability of these approaches.

Here we have reported how the combination of the serum levels of CA 19–9, IGF-1 and albumin using the described algorithm can discriminate between PDAC and CP groups with 93.6% sensitivity and 95% specificity. Some of the markers employed in the combination, in particular albumin and IGF-1, were influenced by the jaundice status of the PDAC patients. CA19.9 levels tended to be elevated in the jaundiced PDAC patients, as previously reported [3, 18], but no significant differences were found between both PDAC groups. Although the absence of jaundiced patients in the CP group is a limitation of the study, the combination of markers still shows a high sensitivity (88.2%) and specificity (90%) to discriminate the non-jaundiced PDAC from the CP patients. The high specificity and sensitivity achieved with this combination has great promise for the differentiation of PDAC from CP, although validation in larger independent cohorts will be required to confirm these data. This combination of biomarkers has the advantage that they can be easily routinely measured in clinical laboratories using autoanalyzers that are already available since the tests used are included in continuous sample access analyzers. Therefore, it could be easily implemented in clinical practice for improving the differential diagnosis of pancreatic cancer.
